# Changes in Magnitude and Variability of Corticospinal Excitability During Rewarded Time-Sensitive Behavior

**DOI:** 10.3389/fnbeh.2019.00147

**Published:** 2019-07-02

**Authors:** Makoto Suzuki, Takako Suzuki, Yin-Jung Wang, Toyohiro Hamaguchi

**Affiliations:** ^1^Faculty of Health Sciences, Tokyo Kasei University, Saitama, Japan; ^2^School of Health Sciences, Saitama Prefectural University, Saitama, Japan

**Keywords:** reward, corticospinal excitability, behavior, schedule, magnetic stimulation

## Abstract

Reward expectation and time estimation are important for behavior and affect corticospinal excitability. This study investigated changes in corticospinal excitability during rewarded time-sensitive behavioral tasks. The rewarded time-sensitive task comprised three fixed-ratio (FR) schedules: FR_A_ contained a reward stimulus after every response, FR_B_ after every two responses, and FR_C_ after every four responses. The participants were instructed to press a left button with the index finger as quickly as possible in response to the appearance of a red circle. Just after the left button press, the word “10-yen” (approximately $0.1) or “no pay” was presented as feedback. Then, the participant had to mentally estimate/wait for 2.5 s from pressing the left button to pressing the right button. One second after the reward stimulus, transcranial magnetic stimulation (TMS) was delivered to the primary motor cortex at the hotspot of the first dorsal interosseous (FDI) muscle. Each participant received items corresponding to the total monetary reward accumulated at the end of the experiment. The variability of motor evoked potential (MEP) amplitudes transformed from a random process during the resting state into an autoregressive process during the rewarded time-sensitive behavioral task. Additionally, the random variation of MEP amplitudes in the FR_C_, FR_B_, and FR_A_ schedules increased in a stepwise fashion. However, the magnitude of MEP amplitudes significantly increased for the FR_B_ and FR_C_ schedules compared to the FR_A_ schedule. The time estimation lag was negative for the three FR schedules but there was no difference among the three FR schedules. The magnitude of corticospinal excitability increased in low reward probability, whereas the variability of corticospinal excitability transformed into an autoregressive process in high reward probability. These results imply that the magnitude and variability of expectation-related corticospinal excitabilities can be differentially altered by reward probability.

## Introduction

The interaction between time estimation and reward perception is crucial to execute behaviors in everyday life. The saying “time flies when we are having fun” refers to how reward influences brain activity during time-sensitive behavior. Previous studies have shown that time estimation and reward perception act by utilizing partially overlapping processing routes (Apaydin et al., 2018). Several brain areas are specialized in temporal processing including the striatum, supplementary motor area, and prefrontal cortex (Bueti et al., 2008; Coull et al., 2011; Üstün et al., 2017; Apaydin et al., 2018), and these brain areas influence M1 activity to execute time-sensitive behavior. Recent studies have indicated that dopamine regulates corticostriatal circuits, and dopamine signaling could modulate time estimation and time-sensitive behaviors (Wiener et al., 2014; Tomasi et al., 2015; Soares et al., 2016).

In human studies, because the corticospinal tract can be activated by transcranial magnetic stimulation (TMS), it has been suggested that the changes in the magnitude and variability of motor evoked potentials (MEPs) depend on M1 activity (Rösler, 2001). Monetary rewards increase MEP amplitudes for the rewarded behavior (Gupta and Aron, 2011; Kapogiannis et al., 2011; Thabit et al., 2011; Borgomaneri et al., 2014; Pisoni et al., 2014; Suzuki et al., 2014), but deprivation of reward as a penalty also increases MEP amplitudes (Suzuki et al., 2018). These observations suggest that reward probability is functionally related to the effectiveness of a reward stimulus, and reward-related signals modulate M1 motor output and MEPs. Especially, a previous study (Nosik and Carr, 2015) indicated that reward probability could momentarily change the value of a consequential reward stimulus, and this phenomenon is termed the “establishing operation.” A previous study on the change in corticospinal excitability during reward tasks indicated that MEP amplitudes before reward stimuli were higher for low reward probability and suggested that this might be related to reward expectation (Suzuki et al., 2014). However, previous studies did not assess the variability of MEP amplitudes but only assessed the magnitude of corticospinal excitability. In addition, previous studies used observational settings without specific behavioral tasks (Kapogiannis et al., 2011; Pisoni et al., 2014) or behavioral tasks unrelated to time perception (Gupta and Aron, 2011; Thabit et al., 2011; Suzuki et al., 2014, 2018). Therefore, it is impossible to know whether expecting a reward or non-reward, based on reward probability, affects the magnitude and variability of corticospinal excitability during time-sensitive behavioral tasks and whether the observed reward-related corticospinal excitability changes are associated with time-sensitive behavioral changes. Therefore, although corticospinal excitability changes are associated with reward expectations, it remains unclear whether reward probabilities affect the magnitude and variability of expectation-related M1 excitability in the context of time-sensitive behavior. These are serious lacunae to elucidate the relationship between reward probability and MEP amplitude changes during time-sensitive behavioral tasks. In addition to expanding on previous findings, exploring how reward probabilities during time-sensitive behavioral tasks affect expectation-related corticospinal excitability may have interesting implications for behavioral science and neuroscience.

Because the temporal resolution of TMS is adequate for observing changes in corticospinal excitability during the rewarded time-sensitive behavioral tasks, we considered that changes in the magnitude and variability of MEPs would be observed using this technique during rewarded time-sensitive behavioral tasks. Therefore, we designed a paradigm involving high and low reward probabilities for time-sensitive behaviors. This paradigm facilitates the investigation of the magnitude and variability of M1 excitability in the context of reward expectation and time estimation. If corticospinal excitability and time estimation change in line with the “establishing operation,” high reward probability contains low reward stimulus value, despite the amount of rewards being large, because high reward probability momentarily decreases the value of a consequential reward stimulus (Nosik and Carr, 2015). In contrast, low reward probability contains high reward stimulus value, despite the amount of rewards being small, because low reward probability momentarily increases the value of a consequential reward stimulus. This raises the question of whether the magnitude and variability of corticospinal excitability related to reward perception reflect the value or the amount of rewards during time-sensitive behavioral tasks. We predicted that if reward amount and value differentially affect M1 excitability, then reward probability should differentially alter the magnitude and variability of MEP amplitudes from the view point of the amount and value of the reward during time-sensitive behavioral tasks. We, therefore, used TMS to investigate expectation-related corticospinal excitation during time-sensitive behavioral tasks with high and low reward probability and to clarify how the magnitude and variability of corticospinal excitations would be altered by reward probability.

## Materials and Methods

### Participants

We recruited 12 healthy participants [eight women and four men, aged 20–21 years, mean ± standard deviation (SD): 20.8 ± 0.4 years] for the behavioral and MEP amplitude measurements. Two participants only took part in the resting state experiments, four participants only in the behavioral experiments, and six participants in both the resting and behavioral experiments described below. No participant had risks of adverse events from TMS (Rossi et al., 2009) or used medication or had any psychiatric or neurological diseases. The Ethics Committee of the Saitama Prefectural University approved the experimental procedures, and the experiments were performed in accordance with the principles of the Declaration of Helsinki. Written informed consent was obtained from all participants.

### Electromyographic (EMG) Recordings

The skin above the first dorsal interosseous (FDI) muscle was cleaned with alcohol to reduce its electrical resistance. Then, double differential surface electrodes (FAD-DEMG1, 4Assist, Tokyo, Japan) adhered on the skin for recording surface EMG activity from the FDI muscle in order to assess corticospinal excitability changes during the rewarded time-sensitive behavioral tasks. The EMG signals were amplified a hundredfold by a DL-140 amplifier (4Assist, Tokyo, Japan), bandpass filtered between 5 and 2,000 Hz and digitized at 10 kHz by a PowerLab system (ADInstruments, Dunedin, New Zealand), and stored on magnetic media.

### TMS

A figure-eight coil (internal diameter of each wing: 70 mm) on the subject’s scalp and a Magstim 200^2^ stimulator (Magstim, Whitland, UK) delivered TMS to the scalp *via* the coil. The coil handle was held approximately 45° to the midline and tangentially to the scalp, thereby a current was induced from the posterolateral to the anteromedial left brain. We determined the appropriate coil position to elicit MEPs in the FDI muscle, and this position was termed the “hotspot” by moving the coil on the left side of the scalp. Then, the hotspot was marked by a soft-tipped pen. The coil was fixed at the hotspot throughout this experiment. The resting motor threshold (RMT) at the hotspot of the relaxed FDI muscle was determined to elicit a MEP of at least 0.05 mV in 5 out of 10 consecutive trials.

### Resting State Experiment

Following excitation of cortical neurons by TMS over the M1, multiple descending volleys are temporally and spatially summated in corticospinal neurons (Rösler, 2001). A previous study (Kiers et al., 1993) noted that MEP amplitudes, shapes, and sizes randomly fluctuated between stimuli. We, therefore, conducted a resting state experiment to confirm the fluctuation of MEP amplitudes. Each participant sat comfortably with their right hand resting on the table throughout the resting state experiment. The MEPs for the FDI muscle were evoked by 20 TMS of 120% of the RMT at the hotspot (the interstimulus interval was 5 s).

### Behavioral Experiment

The behavioral experiment was carried out on a different day from the resting state experiment. Previous experiments using reward tasks (Gupta and Aron, 2011; Thabit et al., 2011; Suzuki et al., 2014, 2018) carried out 18–100 trials per condition. Therefore, the time-sensitive reward task comprised three fixed-ratio (FR) schedules of 50 trials per schedule; the 50 trials of the FR_A_ schedule contained a reward stimulus delivered after every response, the 50 trials of the FR_B_ schedule contained a reward stimulus delivered after every two responses, and the 50 trials of the FR_C_ schedule contained a reward stimulus delivered after every four responses. The order of the three FR schedules was randomized for counterbalancing purposes. The participants were not aware of the reward probabilities and the order of the schedules. The reward probabilities were predetermined.

**Figure 1:**
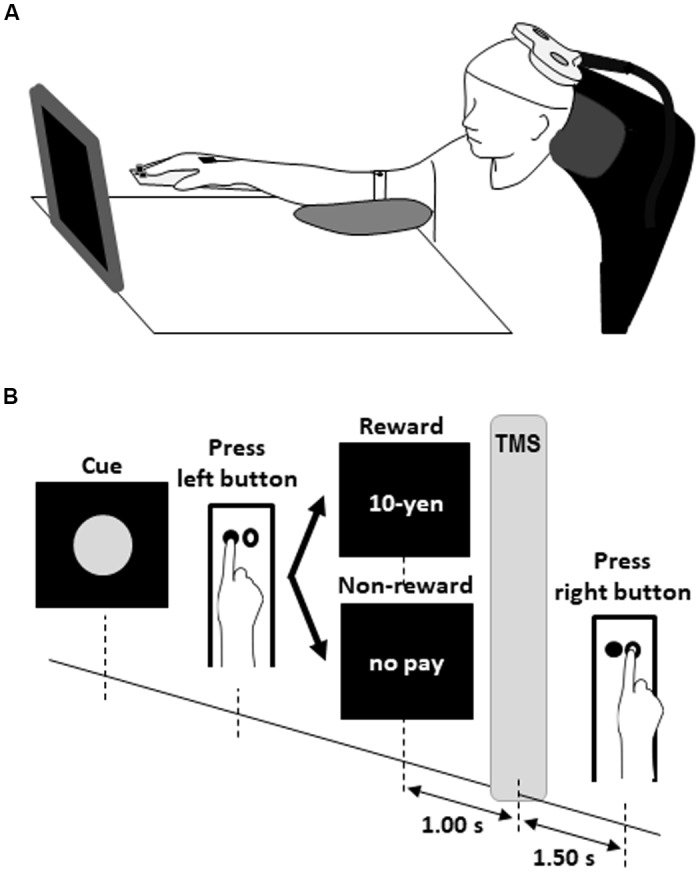
Experimental design for the rewarded time-sensitive behavioral task. Each participant sat comfortably in front of a screen with the right palm and forearm resting on the test equipment with two buttons **(A)**. The left button was pressed with the index finger as quickly as possible after a red circle cue was presented **(B)**. Just after the left button press, the word “10-yen” or “no pay” was presented for 1 s as feedback. The word “10-yen” denoted 10 Japanese yen (approximately $0.1). Then, the participant had to mentally estimate/wait for 2.5 s from pressing the left button to pressing the right button. Single-pulse TMS of 120% of the FDI’s RMT was delivered 1 s after pressing the left button. TMS, transcranial magnetic stimulation; FDI, first dorsal interosseous; RMT, resting motor threshold.

Each participant sat comfortably in front of a 27.5 × 31.0 cm screen located approximately (mean ± SD) 66.9 ± 6.5 cm from the face at 11.3 ± 4.7° downward from the eye level with the right palm and forearm resting on the test equipment with two buttons located 4.0 cm apart parallel to the coronal plane (Figure 1A). The left button was pressed with the index finger as quickly as possible after a red circle cue was presented. The red circle cues were presented on the screen at random intervals of 5–6 s (Figure 1B). The participant was instructed to press the left button with the index finger as quickly as possible in response to the appearance of the red circle. Just after the button press, the word “10-yen” or “no pay” was presented for 1 s as feedback. The word “10-yen” denoted 10 Japanese yen (approximately $0.1). In the FR_A_ schedule, the word “10-yen” or “no pay” would be presented in 100% (50 reward stimuli in 50 presses of the left button) and 0% (zero no-reward stimuli in 50 presses of the left button) of trials, respectively. In the FR_B_ schedule, the word “10-yen” or “no pay” would be presented in 50% (25 reward stimuli in 50 presses of the left button) and 50% (25 no-reward stimuli in 50 presses of the left button) of trials, respectively. In the FR_C_ schedule, the word “10-yen” or “no pay” would be presented in 26% (13 reward stimuli in 50 presses of the left button) and 74% (37 no-reward stimuli in 50 presses of the left button) of trials, respectively. Schultz (2007) noted that dopamine concentrations were greatest at 1 s after the presentation of a reward stimulus and returned to baseline after approximately 4 s. Borgomaneri et al. (2012) noted that corticospinal excitability increased at least 300 ms after the presentation of pictures representing negative emotion. Thabit et al. (2011) noted that corticospinal excitability increased 1 s after the presentation of a reward stimulus for 3- to 4-s intervals. We set the delivery time of TMS and inter-trial interval in our protocol in consideration of the previous studies’ time courses and delivered TMS of 120% of the FDI’s RMT 1 s after pressing the left button. Then, the participant had to mentally estimate/wait for 2.5 s from pressing the left button to pressing the right button. Therefore, 50 TMSs were delivered in each FR schedule because the participants pressed the left button iteratively 50 times after the reward or no-reward stimulus. This ensured that the magnitude and variability of corticospinal excitability reflected the expectation of reward or non-reward during time-sensitive behavioral tasks. Each participant received items corresponding to a total of 870 Japanese yen (approximately $8.7) as reward accumulated at the end of the experiment.

### Data Analysis

To facilitate investigations of intraindividual MEP variability during the time-sensitive reward task, the MEP data were normalized by linear transformation. The normalized MEP data are expressed as Z scores. We predicted that TMS over the M1 would naturally induce a random fluctuation of MEP amplitudes and that time-oriented reward perception would transform activity of the M1 *via* corticospinal excitability from a random process into an autoregressive process because the autoregressive process could indicate that the MEP amplitude was affected not by random fluctuation but by the preceding MEP amplitudes related to reward or no-reward stimuli from the previous trials. Therefore, a state-changing model was constructed, which included trend, autoregressive, and random fluctuation processes to distinguish between inherent MEP changes by the reward stimulus and MEP random fluctuation as follows:

(1) $$f(t)=\alpha +\beta t+\sum_{i=1}^{p}\phi_{i}x_{t-i}+\varepsilon_{t}$$

where *α* is the y-intercept of the MEP amplitude, reflecting initial corticospinal excitability; *β* is the MEP amplitude slope, reflecting changes in corticospinal excitability; *φ* and *x* are the coefficient and previous reference MEP amplitudes of the autoregressive model, reflecting the temporal dependance structure of a time series; *ε*_t_ is the random variation, reflecting the inherent fluctuation of MEPs; *i* is the order of the model, and *t* is the number of TMS deliveries during the time-sensitive reward task. By the least-squares method, each participant’s data were fitted to the model. If the model is applicable, the series of values of *ε*_t_ in Equation (1) should be uncorrelated to each other (i.e., independence). Therefore, we assessed the applicability of the model with the Ljung–Box test to measure the independence of *ε*_t_ as a white noise and residuals process. The following equation was used for the Ljung–Box test.

(2) $$Q(h)=n(n+2)\sum_{i=1}^{h}\frac{{\hat{\rho }}_{i}{}^{2}}{n-i}$$

where *n* is the sample size (${\hat{\rho }}_{i}$) is the sample autocorrelation at lag *i*, and *h* is the number of lags being tested. Thus, the data eliminate inherent fluctuations of MEPs, permitting the evaluation of whether reward probability affects corticospinal excitability during time-sensitive behavioral tasks. Differences in the MEP amplitudes eliminating inherent fluctuations between three FR schedules and 50 trials were compared by two-way repeated measures analysis of variance (ANOVA). *Post hoc* testing with Bonferroni correction was performed to compare differences in MEP amplitudes among the three FR schedules. We also compared the MEP amplitudes across trials following presentation of the word “10-yen” or “no-pay” to assess the effect of the immediately preceding reward or no-reward stimulus on expectation-related corticospinal excitability by unpaired *t*-test. Moreover, the permutated Brunner–Munzel test was performed to carefully assess intra- and inter-individual changes for small sample data because the asymptotic permutational distribution of this test using the central limit theorem can deduce the standard normal distribution and accurate *p*-value (Fagerland et al., 2011). Response time was calculated as the elapsed time between the left and the right button presses. The time lag between the absolute target time (2.5 s) and subjective response time (the elapsed time between the left and right button presses) was calculated for each trial for each participant to predict change in the participant’s time estimation. To assess group changes, we compared time estimation data based on the response time across the FR schedules using one-way ANOVA. In addition, we compared the time estimation lag across trials immediately preceding a reward (“10-yen”) or no-reward (“no-pay”) stimulus by unpaired *t*-test and the permutated Brunner–Munzel test. We defined statistical significance as *p* < 0.05. All statistical analyses were performed with R 3.4.0 software (R Foundation for Statistical Computing, Vienna, Austria).

## Results

No participant had adverse TMS-related effects in any experiment.

### Corticospinal Excitability During the Resting State

**Figure 2:**
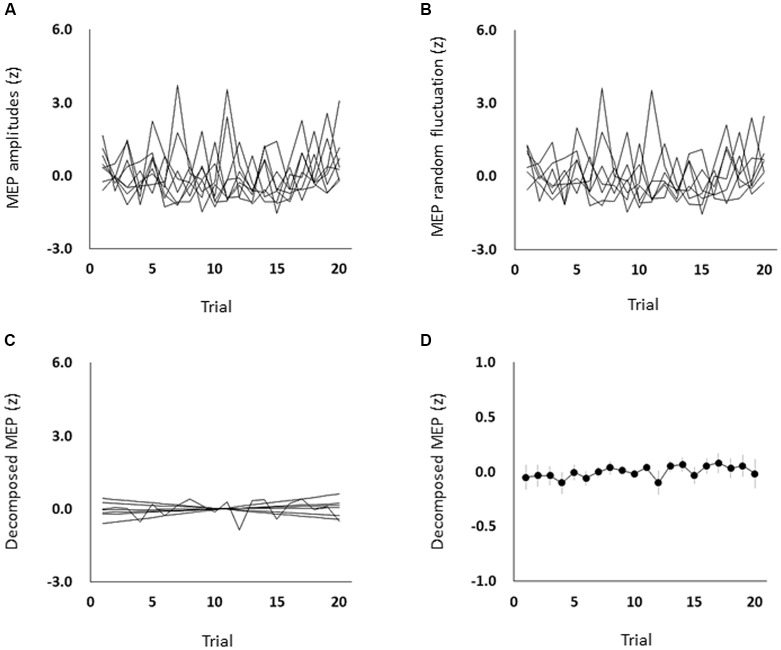
The time course of changes in each participant’s MEP amplitudes **(A)**, each participant’s MEP random fluctuation **(B)**, each participant’s MEP amplitudes eliminating random fluctuation **(C)**, and the mean MEP amplitudes eliminating random fluctuation **(D)** during the resting state. The symbols and error bars denote means and standard errors of the mean, respectively. Raw MEP amplitude changes across trials were jumbled, whereas MEP amplitudes eliminating random fluctuations were generally stable. MEP, motor-evoked potential.

The mean ± standard errors of MEP amplitudes of the FDI muscle during the resting state was 0.94 ± 0.06 mV. Figure 2A shows the time course of changes in FDI MEP amplitudes in the resting state. Figures 2B,C show the random fluctuation of MEP amplitudes [*ε*_t_ value in Equation (1)] and the MEP amplitudes eliminating inherent random fluctuations, respectively. Table 1 shows the *α*, *β*, *p*, and *φ* values in Equation (1) for the resting state. Two of eight (25.0%) participants’ *α* values were positive, and six of eight (75.0%) participants’ *α* values were negative. However, six of eight (75.0%) participants’ *β* values were positive and two of eight (25.0%) participants’ *β* values were negative. Figure 2D shows the time-series plots of the decomposed mean MEP amplitudes during the resting state. Figure 2A indicates that the raw MEP amplitude increases and decreases during trials, whereas Figures 2C,D indicate that the MEP amplitudes eliminating inherent fluctuations (*ε*_t_) were generally stable. Based on the *p* parameter estimation of Equation (1), in seven of eight (87.5%) participants, the *p*-value of the model was 0, which indicates that the errors were uncorrelated across time. In one of eight (12.5%) participants, the *p*-value of the model was 1, indicating an autoregressive process with a 1-bin time lag and that previous corticospinal excitability affected the variability of corticospinal excitability. The Ljung–Box test showed that the series of *ε*_t_ of the model was independent in eight of eight (100%) participants, which indicates that the model was efficient.

**Table 1:** Assessment of the model fit in the resting state experiment.

Participants	Trend term		AR term		Box-Ljung test	
	*α*	*β*	*p*^#^	*φ*	*χ*^2^	p*
1	−0.25	0.02	0	-	0.64	0.43
2	−0.67	0.06	0	-	0.18	0.67
3	−0.02	0.00	1	−0.3569	2.82	0.09
4	−0.17	0.02	0	-	0.08	0.77
5	0.29	−0.03	0	-	0.30	0.59
6	0.48	−0.05	0	-	0.41	0.52
7	−0.05	0.00	0	-	1.56	0.21
8	−0.05	0.00	0	-	0.34	0.56
Total	−0.13 ± 0.10	0.01 ± 0.01				

MEP, motor evoked potential; FR, fixed-ratio; AR, autoregressive. ^#^*p* value of the Equation (1). *p value of the Ljung-Box test.

### Corticospinal Excitability During the Time-Sensitive Behavioral Tasks

**Figure 3:**
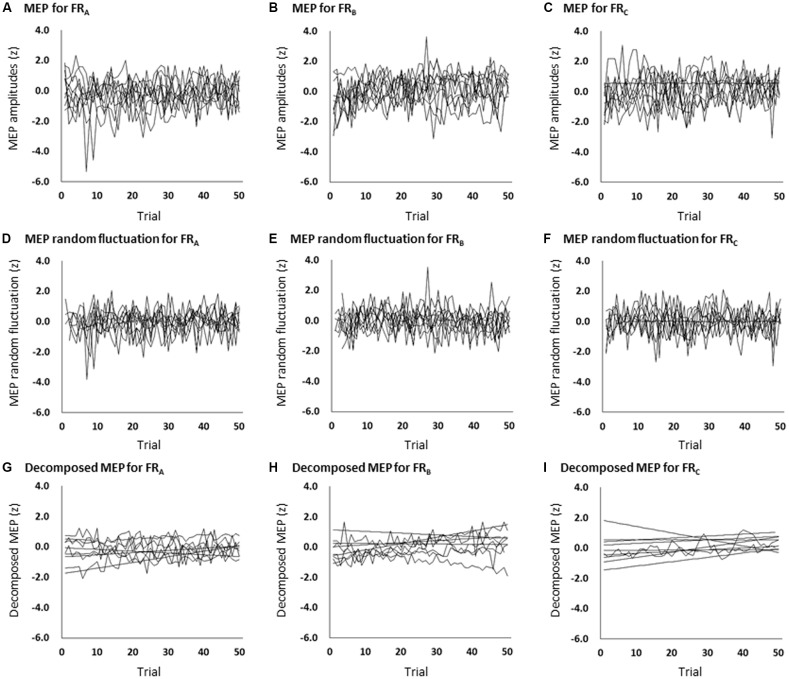
The time course of changes in each participant’s MEP amplitudes during the FR_A_ **(A)**, FR_B_ **(B)**, and FR_C_ **(C)** schedules; each participant’s MEP random fluctuation during the FR_A_ **(D)**, FR_B_ **(E)**, and FR_C_ **(F)** schedules; and each participant’s MEP amplitudes eliminating random fluctuation during the FR_A_ **(G)**, FR_B_ **(H)**, and FR_C_ **(I)** schedules. The slopes (*β* values) were higher for the FR_B_ and FR_C_ schedules than for the FR_A_ schedule. MEP, motor-evoked potential; FR, fixed-ratio.

**Figure 4:**
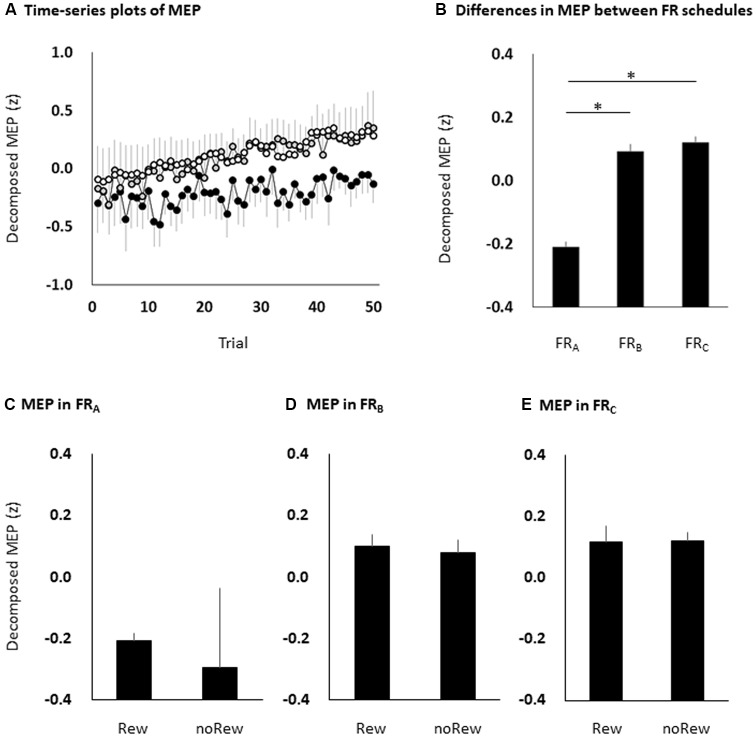
The time course of changes in MEP amplitudes eliminating random fluctuation in the three FR schedules **(A)**, bar graphs of MEP amplitudes to pool the data for the three FR schedules **(B)**, and the MEP amplitudes of the immediately preceding reward or no-reward stimulus in the FR_A_ **(C)**, FR_B_ **(D)** and FR_C_ **(E)** schedules. The black (FR_A_), grey (FR_B_) and white (FR_C_) symbols and error bars **(A)** denote means and standard errors of the mean, respectively. The columns and error bars **(B–E)** denote the means and standard errors of the mean, respectively. Two-way repeated measures ANOVA showed that there was no significant interaction effect in the three FR schedules and 50 trials (*F* = 0.267, *p* = 0.769). *Post hoc* Bonferroni correction showed that the MEP amplitudes obtained for the FDI muscle significantly increased for the FR_B_ and FR_C_ schedules compared to the FR_A_ schedule (FR_A_ vs. FR_B_, *p < 0.0001; FR_A_ vs. FR_C_, *p < 0.0001; FR_B_ vs. FR_C_, *p* = 1.000; **B**). However, there were no significant differences in MEP amplitudes immediately preceding the reward or no-reward stimulus in any FR schedule (unpaired *t*-tests, FR_A_, *p* = 0.746; FR_B_, *p* = 0.758; FR_C_, *p* = 0.969; **C–E**). MEP, motor-evoked potential; FR, fixed-ratio; ANOVA, analysis of variance; Rew: presentation of reward (“10-yen”) in the immediately preceding trial; noRew: presentation of no-reward (“no-pay”) in the immediately preceding trial.

All subjects completed all experimental conditions. Erroneous button presses did not occur during the experiments. Table 2 shows the MEP amplitudes obtained from the FDI muscle during the three FR schedules. Figure 3 shows the time courses of changes in FDI MEP amplitudes during the three FR schedules. Table 3 shows the differences in *α*, *β*, *p*, and *φ* values for the three FR schedules. The *α* values were almost the same across the three FR schedules; 4 of 10 (40.0%) participants’ *α* values were positive for the FR_A_ schedule, five of 10 (50.0%) participants’ *α* values were positive for the FR_B_ schedule, and 4 of 10 (40.0%) participants’ *α* values were positive for the FR_C_ schedule. However, the *β* values were higher for the FR_B_ and FR_C_ schedules than for the FR_A_ schedule; five of 10 (50.0%) participants’ *β* values were positive for the FR_A_ schedule, seven of 10 (70.0%) participants’ *β* values were positive for the FR_B_ schedule, and eight of 10 (80.0%) participants’* β* values were positive for the FR_C_ schedule. Figure 4A shows the time-series plots of the decomposed mean MEP amplitudes during the rewarded time-sensitive behavioral tasks. Two-way repeated measures ANOVA showed that there was no significant interaction effect in the three FR schedules and 50 trials (*F* = 0.267, *p* = 0.769). This allowed us to pool the MEP amplitudes measured from the FDI muscle in the three FR schedules. *Post hoc* Bonferroni correction showed that the MEP amplitudes obtained for the FDI muscle significantly increased for the FR_B_ and FR_C_ schedules compared to the FR_A_ schedule (FR_A_ vs. FR_B_, *p* < 0.0001; FR_A_ vs. FR_C_, *p* < 0.0001; FR_B_ vs. FR_C_, *p* = 1.000; Figure 4B). In addition, the permutated Brunner–Munzel test also showed that the MEP amplitudes for the FDI muscle in the FR_B_ and FR_C_ schedules were significantly greater than those in the FR_A_ schedule (FR_A_ vs. FR_B_, *p* < 0.0001; FR_A_ vs. FR_C_, *p* < 0.0001), but no such difference was observed between the FR_B_ and FR_C_ schedules (*p* = 0.812; Figure 4B). However, unpaired *t*-tests showed that there were no significant differences in MEP amplitudes immediately preceding the reward (“10-yen”) or no-reward (“no-pay”) stimulus in any FR schedule (FR_A_, *p* = 0.746; FR_B_, *p* = 0.758; FR_C_, *p* = 0.969; Figures 4C–E). The permutated Brunner–Munzel test also showed that there were no significant differences in MEP amplitudes immediately preceding the reward (“10-yen”) or no-reward (“no-pay”) stimulus in any FR schedule (FR_A_, *p* = 0.925; FR_B_, *p* = 0.617; FR_C_, *p* = 0.986). Based on the *p* parameter estimation of Equation (1), a 0 *p*-value was more frequent in the FR schedules of lower reward probability; three of 10 (30.0%) participants’ *p*-values were 0 in the FR_A_ schedule, 4 of 10 (40.0%) participants’ *p*-values were 0 in the FR_B_ schedule, and seven of 10 (70.0%) participants’ *p*-values were 0 in the FR_C_ schedule. The Ljung–Box test showed that the series of *ε*_t_ values in Equation (1) was independent in 10 of 10 (100%) participants for the three FR schedules.

**Table 2:** MEP amplitudes corresponding to the FR schedules.

	MEP amplitudes (mV)		
Subjects	FR_A_	FR_B_	FR_C_
1	1.51 ± 0.03	1.63 ± 0.04	1.63 ± 0.04
2	3.99 ± 0.20	2.80 ± 0.15	2.39 ± 0.00
3	5.30 ± 0.12	6.63 ± 0.19	4.47 ± 0.14
4	1.30 ± 0.05	0.74 ± 0.03	1.13 ± 0.04
5	3.07 ± 0.20	2.08 ± 0.13	2.84 ± 0.20
6	4.40 ± 0.15	2.88 ± 0.28	3.88 ± 0.27
7	1.00 ± 0.08	1.27 ± 0.09	1.14 ± 0.07
8	1.21 ± 0.07	0.66 ± 0.04	0.60 ± 0.04
9	3.03 ± 0.06	3.09 ± 0.07	2.11 ± 0.14
10	1.77 ± 0.11	2.29 ± 0.21	3.66 ± 0.18
Total	2.65 ± 0.08	2.41 ± 0.09	2.38 ± 0.07

Values are mean ± standard error of the mean. MEP, motor-evoked potential; FR, fixed-ratio

**Table 3:** Assessment of the model fit.

	Trend term		AR term		Box-Ljung test	
Subjects	*α*	*β*	*p*^#^	*φ*	*χ*^2^	p*
**A. FR_A_ schedule**						
1	0.76	−0.017	4	0.42, −0.32, 0.31, −0.28	3.85	0.050
2	−1.43	0.027	5	0.23, −0.08, −0.25, 0.33, −0.29	0.42	0.519
3	0.27	−0.006	2	−0.12, −0.36	0.40	0.527
4	−0.41	−0.010	1	0.28	0.04	0.850
5	−0.67	0.014	0	-	0.14	0.707
6	−0.49	0.005	8	−0.26, −0.19, −0.23, −0.19, 0.08, −0.15, −0.25, −0.32	0.75	0.388
7	0.53	−0.011	8	0.15, −0.11, 0.14, 0.37, 0.04, −0.14, −0.13, −0.32	2.85	0.091
8	−1.78	0.037	0	-	0.18	0.674
9	−0.06	−0.012	0	-	1.11	0.291
10	0.29	0.010	1	0.22	2.53	0.112
Total	−0.30 ± 0.26	0.00 ± 0.01				
**B. FR_B_ schedule**						
1	−0.50	0.013	2	0.19, 0.33	0.04	0.850
2	0.25	−0.002	0	-	0.20	0.654
3	0.00	−0.032	1	−0.23	2.92	0.087
4	1.15	−0.011	0	-	1.54	0.215
5	0.43	0.001	2	−0.15, 0.23	1.92	0.166
6	−0.60	0.042	0	-	0.78	0.378
7	−0.82	0.023	1	0.25	3.17	0.075
8	0.02	0.014	0	-	0.06	0.807
9	−0.63	0.008	2	−0.16, −0.33	0.69	0.405
10	−1.11	0.051	3	0.27, −0.31, 0.31	1.23	0.267
Total	−0.18 ± 0.21	0.01 ± 0.01
**C. FR_C_ schedule**						
1	−0.47	0.012	0	-	0.65	0.420
2	0.55	−0.0002	5	0.04, 0.14, 0.16, −0.31, 0.20	0.24	0.626
3	0.40	0.013	0	-	0.70	0.404
4	−0.36	0.007	1	0.23	2.73	0.098
5	−0.15	0.0004	0	-	1.08	0.300
6	−0.94	0.034	0	-	0.12	0.725
7	−0.63	0.024	7	0.12, −0.03, −0.19, −0.27, −0.13, −0.15, −0.27	0.03	0.856
8	0.19	0.012	0	-	0.01	0.943
9	1.86	−0.043	0	-	0.57	0.449
10	−1.48	0.03	0	-	0.06	0.804
Total	−0.10 ± 0.29	0.01 ± 0.01				

MEP: motor evoked potential; FR: fixed-ratio; AR: autoregressive. ^#^*p* value of the Equation (1). *p value of the Ljung-Box test.

### Time Estimation During the Time-Sensitive Behavioral Tasks

**Figure 5:**
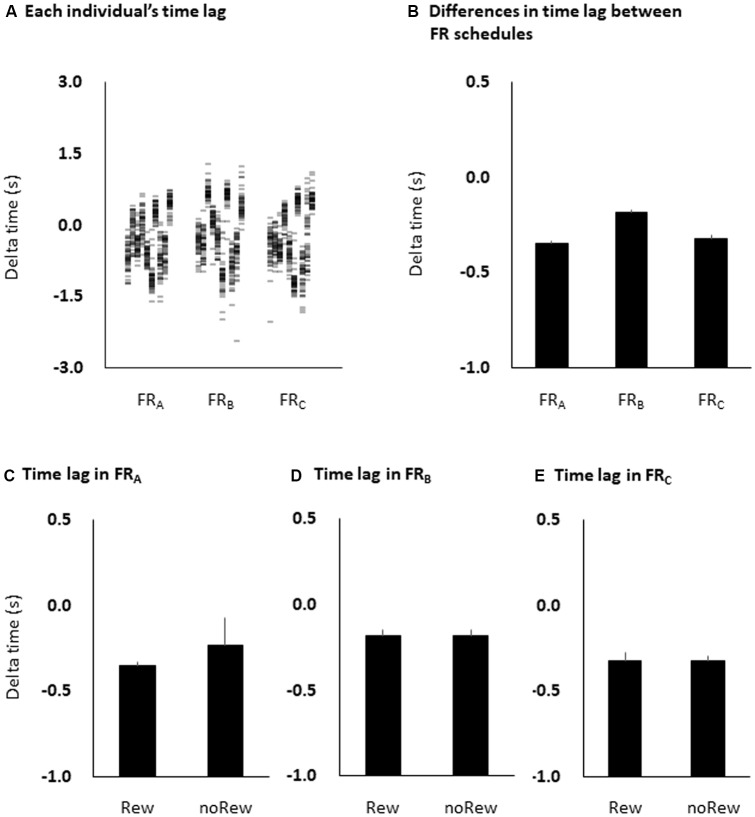
Time estimation lags among the three FR schedules at the individual level **(A)** and the group level **(B)**, and among the immediately preceding reward or no-reward stimulus in the FR_A_ **(C)**, FR_B_ **(D)** and FR_C_ **(E)** schedules. The columns and error bars denote the means and standard errors of the mean, respectively. The difference between time estimation lags observed among the three FR schedules was small and non-significant (one-way ANOVA, *F* = 0.458, *p* = 0.499; **B**), although the time estimation lags were negative in all three FR schedules. Additionally, there were also no significant differences in time estimation lag immediately preceding the reward (“10-yen”) or no-reward (“no-pay”) stimulus in any FR schedule (unpaired *t*-tests, FR_A_, *p* = 0.483; FR_B_, *p* = 0.964; FR_C_, *p* = 0.992; **C–E**). FR, fixed-ratio; ANOVA, analysis of variance.

The time lag between absolute target time and subjective response time was −0.35 ± 0.02 ms for the FR_A_ schedule, −0.18 ± 0.03 ms for the FR_B_ schedule, and −0.32 ± 0.03 ms for the FR_C_ schedule. Although the time lag was negative in all three FR schedules, one-way ANOVA showed that there were no significant differences among the FR schedules (*F* = 0.458, *p* = 0.499; Figures 5A,B). Additionally, unpaired *t*-tests showed that there were no significant differences in the time estimation lag immediately preceding the reward (“10-yen”) or no-reward (“no-pay”) stimulus in any FR schedule (FR_A_, *p* = 0.483; FR_B_, *p* = 0.964; FR_C_, *p* = 0.992; Figures 5C–E). The permutated Brunner–Munzel test also showed that there were no significant differences in MEP amplitudes immediately preceding the reward (“10-yen”) or no-reward (“no-pay”) stimulus in any FR schedule (FR_A_, *p* = 0.384; FR_B_, *p* = 0.982; FR_C_, *p* = 0.894).

## Discussion

To test the hypothesis that reward amount and value should differentially affect the magnitude and variability of corticospinal excitability, we measured changes in the magnitude and variability of the MEP amplitude related to reward expectation during a time-sensitive behavioral task. Our results showed that: (a) the variability of expectation-related MEP amplitudes transformed from a random process during the resting state into an autoregressive processes during the time-sensitive behavioral task; (b) the random variation of MEP amplitudes in the FR_C_, FR_B_, and FR_A_ schedules decreased in a stepwise fashion; (c) the magnitude of the MEP amplitudes increased for the FR_B_ and FR_C_ schedules compared to the FR_A_ schedule; and (d) the time estimation lag was negative for and similar among the three FR schedules. These observations show that reward probability modulated M1 motor output and MEPs. In fact, although the magnitude of the MEP amplitudes was higher in low reward probability (FR_C_ schedule) than in high reward probability (FR_A_ schedule), the variability of the MEP amplitudes was transformed into a time-varying autoregressive process by high reward probability (FR_A_ schedule) rather than by low reward probability (FR_C_ schedule). This implies that reward probability does not equally affect the magnitude and variability of corticospinal excitability. To our knowledge, this is the first systematic study to report that reward probabilities change the magnitude and variability of expectation-related corticospinal excitabilities during time-sensitive behavior.

Many areas including the ventral tegmental area, striatum, supplementary motor area, and prefrontal cortex influence M1 activity in terms of reward processing (Wickens et al., 2003; Haruno et al., 2004; Campos et al., 2005; Ikemoto, 2007; Hikosaka et al., 2008). In addition, similar brain areas are also specialized in temporal processing including the striatum, supplementary motor area, and prefrontal cortex (Bueti et al., 2008; Macdonald et al., 2012; Failing and Theeuwes, 2016; Apaydin et al., 2018). Dopamine neurons connect to the striatum and prefrontal cortex (Haber and Knutson, 2010; Averbeck et al., 2014; Haber, 2016). In addition, the prefrontal cortex connects to the supplementary motor area (Goldman-Rakic, 1987); thus, prefrontal input is provided from dopamine neurons to the supplementary motor area, which in turn connects to the M1. Moreover, a retrograde tracing study found that approximately 70% of dopamine neurons in the midbrain projected to the M1 (Hosp et al., 2011). Previous studies have suggested that bursts of dopaminergic activity in the midbrain serve as time perception (Soares et al., 2016). These previous findings regarding neural networks and physiological mechanisms suggested that overall coactivation of the corticostriatal circuit including the ventral tegmental area, striatum, supplementary motor area, and prefrontal cortex might reveal the time perception and reward processing through direct and indirect projections of dopaminergic and glutamatergic neurons, and these circuits may influence corticospinal excitability *via* the M1. In our study, TMS was delivered 1 s after the presentation of reward or no-reward stimuli in accordance with the previous studies’ time courses regarding dopamine concentration and corticospinal excitation by reward presentation (Schultz, 2007; Thabit et al., 2011). This experimental setup allowed us to investigate changes in the magnitude and variability of MEPs during rewarded time-sensitive behavioral tasks. In our study, the magnitudes of the MEP amplitudes before reward presentation increased for low reward probability. This is the first novel observation of our study. Although the exact mechanism for high MEP amplitudes for low reward probability were not identified, we predict that M1 excitability during the time-sensitive behavioral task could have been influenced by reward probability. One possibility is that the activities of many brain regions, including the ventral tegmental area, striatum, supplementary motor area, and prefrontal cortex may affect M1 activity with different gains according to reward probability. Especially, recent research findings have suggested that low reward probability, rather than high reward probability, increases the number of behaviors (Derosa et al., 2015; Fisher et al., 2018). This phenomenon termed the “establishing operation” occurs as a result of low reward probability momentarily increasing the value of a consequential reward stimulus (Derosa et al., 2015; Nosik and Carr, 2015; Fisher et al., 2018). In addition, previous studies have suggested that low reward probabilities (Suzuki et al., 2014), upsetting images (Oliveri et al., 2003; Coelho et al., 2010; Borgomaneri et al., 2012), and unexpected penalties also increase corticospinal excitability (Suzuki et al., 2018). These may imply that M1 excitation may increase in line with the “establishing operation” or with no-reward in low reward probability. However, the MEP amplitudes immediately preceding the reward (“10-yen”) or no-reward (“no-pay”) stimulus did not differ in any of the three FR schedules. Therefore, changes in M1 excitability related to reward probability might be affected by the global reward signal throughout each FR schedule. To clarify this, further research is needed on the time course of changes in M1 excitability in relation to various reward settings, including rewards and penalties, in fixed- and variable-ratio schedules.

Kiers et al. (1993) studied the variability of MEPs produced by TMS and noted that the variability in MEPs is essentially random in the resting state. In our study, the *p*-value of the model was 0 in most datasets during the resting state, which indicates that the variability of the MEP amplitudes was uncorrelated across time and a random process. However, TMS-evoked MEP amplitude variability was a time-varying autoregressive process during the time-sensitive behavioral task. In addition, the random variability of MEP amplitudes decreased from low reward probability (i.e., FR_C_) to high reward probability (i.e., FR_A_). This is the second novel observation of our study. It has been previously noted that the frontal network was engaged in time perception, reward perception, and working memory (Üstün et al., 2017; Apaydin et al., 2018). In our study, the participant waited for 5–6 s until seeing the next reward stimulus in the FR_A_ schedule, whereas the participant waited for 20–24 s until seeing the next reward stimulus in the FR_C_ schedule. This interval of reward presentation may affect the variability of corticospinal excitabilities during time-sensitive behavioral tasks from the standpoint of memory retention time. In fact, the red circle cue did not indicate reward signals and schedules but only preannounced reward appearance. Therefore, the subjects might expect the reward in reference to the history of reward appearances. Hence, our findings showed that high reward probability facilitates the variability of expectation-related M1 excitability in an autoregressive manner, which extends the results of previous studies and supports the proposition that reward probability affects the variability of expectation-related corticospinal excitability.

In this study, the time estimation lag was negative in all three FR schedules. Soares et al. (2016) found that activation or inhibition of dopamine neurons contributed to decelerate or accelerate time estimation, respectively. Our result suggests that reward may decelerate time estimation and delay response time, and consequently, the time estimation lag became negative. However, there were no differences in the time estimation lag among the three FR schedules. Additionally, the time estimation lag immediately preceding the reward (“10-yen”) or no-reward (“no-pay”) stimulus did not differ in any of the three FR schedules. In previous reward tasks (Kapogiannis et al., 2008; Gupta and Aron, 2011; Thabit et al., 2011; Suzuki et al., 2014, 2018), 10–500 Japanese yen (approximately $0.1 to $5) were used as a monetary reward. However, in previous penalty tasks (Suzuki et al., 2018), the penalty stimulus indicated that the participant lost 100 Japanese yen (approximately $1.0). In our study, the reward stimulus was the word “10-yen,” which had a rewarding value as it represented 10 actual Japanese yen. The non-reward stimulus was the word “no-pay,” which did not have rewarding value. Therefore, the stimulus gap between “10-yen” and “no-pay” may be too small to clarify the changes in the time estimation lag among the three FR schedules. In the context of the gap between reward and no-reward, a higher reward may emphasize changes in the time estimation lag during time-sensitive behavioral tasks. In our study, the participant had to mentally estimate/wait for 2.5 s after TMS with suprathreshold intensity. Although previous studies suggested that TMS delays or shortens the reaction time according to the intensity of the stimuli (Pascual-Leone et al., 1992a,b), a 2.5 s waiting time is sufficiently long to reduce the effect of TMS on reaction time. Therefore, the effect on the time estimation lag of TMS in this study was considered minimal. However, the role of changes in corticospinal excitability during time-sensitive behavioral tasks for decelerating time estimation remains unclear. Further research is needed to investigate the relationship between the time estimation process and corticospinal excitability using higher reward stimuli.

A potential limitation of our study is the small sample size, although the permutated Brunner–Munzel test can deduce the standard normal distribution and accurate *p*-value in small sample data (Fagerland et al., 2011). In addition, corticostriatal neuronal activities related to midbrain dopaminergic neurons could not be directly observed. A previous study (Fiorillo et al., 2003) suggested that reward and penalty outcomes are related to the firing of dopaminergic neurons. A study by Koepp et al. (1998) found evidence that dopamine was released in the human striatum during a behavioral task. Another study (Zald et al., 2004) noted that rewards increased dopamine transmission. A larger number of participants will be needed in future studies, and additional detailed examination using both TMS and brain imaging methods should be conducted to identify the neuronal effects of reward probabilities.

In conclusion, we found that reward probabilities were associated with expectation-related corticospinal excitabilities during a time-sensitive behavioral task. In fact, the magnitude of corticospinal excitability increased in low reward probability, whereas the variability of corticospinal excitability transformed into an autoregressive process in high reward probability. These results imply that the magnitude and variability of corticospinal excitabilities can be differentially altered by reward probability. These findings have implications for the characteristics of corticospinal excitation including M1 changes during rewarded time-sensitive behavior.

## Ethics Statement

The experimental procedures were approved by the Ethics Committee of the Saitama Prefectural University and performed in accordance with the principles of the Declaration of Helsinki. All participants provided written informed consent.

## Author Contributions

MS participated in the design of the study, carried out the experiment, performed the statistical analyses, and drafted the manuscript. TS conceived the study, participated in its design, carried out the experiment, and drafted the manuscript. Y-JW conceived the study, participated in its design, and helped with the experiment. TH conceived the study, participated in its design, and drafted the manuscript. All authors read and approved the final manuscript.

## Conflict of Interest Statement

The authors declare that the research was conducted in the absence of any commercial or financial relationships that could be construed as a potential conflict of interest.

## Abbreviations

FDI: first dorsal interosseous
FR: fixed ratio
MEP: motor evoked potential
RMT: resting motor threshold
TMS: transcranial magnetic stimulation.
